# The Lesson of the Metropolitan Asylums Board Report

**Published:** 1914-09-26

**Authors:** 


### POOR-LAW SICK RELIEF IN LONDON.

### The Lesson of the Metropolitan Asylums Board Report.

During the year under review in the recently issued Report of the Metropolitan Asylums Board, some specific points in which were touched on in a recent number of The Hospital, the most important new work undertaken by the Board has been the provision of sanatoria for insured tuberculous patients. The Downs Sanatorium, Sutton, was opened early in the year with 350 beds, and later 200 beds at the Northern Hospital, Winchmore Hill, were set apart for the same class of patients. More recently an agreement has been entered into with the London County Council by which the Board is to erect two sanatoria for men and one for women. As regards chronic cases, St. George's Home, Milman Street, has been opened for advanced cases of tuberculosis in women; but the Board rightly points out that the provision of special buildings for the 2,300 cases at present housed in Poor-Law infirmaries could not be justified. The arrangements for the treatment of uninsured persons are now under discussion with the County Council.

### The Success of Centralisation.

Another matter of very great importance has been the success of the policy of the Casual Ward Committee in reducing the number of vagrant poor. In 1910 there were 1,082 persons in the casual wards of London on the last Friday in December. On the same day in 1913 there were only 228 (204 men, 23 women, and 1 child). The committee points out that there has been no increase in the number of inmates of casual wards in areas adjacent to London, and that 570 applicants in 1913 were assisted without having to enter the wards at all, and that many of them were enabled to return to the normal life of self-supporting citizens. One result of this reduction in the tramp population has been that of the twenty-eight casual wards in existence prior to the transfer to the Board in 1912 it was found necessary to keep open only twelve at the end of 1913. Turning to the older work of the Board it is found that the number of insane for whom it is responsible still continues to increase steadily. On January 1, 1913, there were 7,709 inmates in its mental institutions. During the year 1,078 patients were admitted, and on December 31, 1913, there were remaining 7,844. The pressure on the accommodation was such that the admission of patients was delayed, and sometimes considerably delayed. To meet the demands upon it the Board has decided to double the accommodation at the Tooting Bec institution, and to increase the beds there from 1,114 to 2,220. As regards fever patients, the Board had to face during the year an epidemic of scarlet fever of a mild type, but involving the admission of 5,127 patients more than in the previous year. To meet this increase the Board had to limit its admissions of cases of measles and whooping-cough and to arrange for the transference of the Fountain Hospital, which had been used for imbecile children, back to the infectious service. To provide for the imbecile children which will thus be displaced, the Board took over in April 1914 the Edmonton Workhouse of the City of Westminster Guardians.

In the children's service attention is drawn to the excellent results achieved at East Cliff House, Margate, in cases of children suffering from tuberculous disease of the joints, bones, and glands. The reconstruction and enlargement of this institution from 140 to 270 beds are now being proceeded with. Open-air verandahs form a prominent part of this reconstruction, and instead of the verandah being an adjunct to the ward, the ward will be only an adjunct of the verandah, and by far the larger number of cases will receive verandah treatment pure and simple. The Board anticipates that an extension of Queen Mary's Hospital, Carshalton, will soon be necessary to meet the requirements of accommodation for tuberculous children and also to enable the Park Hospital to revert to the infectious service when necessary. (See The Hospital, July 26, 1913.)

The number of patients admitted to the various hospitals and asylums of the Board in the year is enormous; 27,746 cases of infectious disease were admitted, 1,078 mental cases, 1,922 tuberculous patients, 4,255 sick and convalescent children, 754 ringworm children, 459 children suffering from ophthalmia, and 46,670 to the casual wards.

### Preventive Work and the Moral.

The only important additions to the staff during the year were the appointment of Dr. J. Galloway as consulting physician for skin diseases to the children's hospitals, and of Dr. William Mair as research pathologist for a period of one year in the first instance. The question of the appointment of an otologist with a view to the prevention of deafness following infectious disease was considered by the Hospitals Committee, but for reasons not stated the committee decided not to take any action in the matter. This decision is to be regretted, and it is to be hoped the matter will be reconsidered in the near future. There is a very important field for preventive work in this direction, and the good which could be done would amply repay the cost.

As regards finances the Board is able to dwell with satisfaction upon the large permanent reduction effected in recent years by improved methods of organisation which have greatly diminished the cost per patient maintained, and especially upon the fact that under its scheme for the consolidation of its loans the amount paid in interest will be £300,000 less than it otherwise would have been, and that in eight years the whole of the existing loans and loan charges will be paid off.

In reading this Report one cannot help feeling how much the administration of Poor-Law sick relief in the Metropolis might be simplified and improved if the whole of it could be amalgamated under one central body such as an enlarged Metropolitan Asylums Board.

